# LXW7 ameliorates focal cerebral ischemia injury and attenuates inflammatory responses in activated microglia in rats

**DOI:** 10.1590/1414-431X20165287

**Published:** 2016-08-01

**Authors:** T. Fang, D. Zhou, L. Lu, X. Tong, J. Wu, L. Yi

**Affiliations:** Department of Neurology, Shenzhen Hospital, Peking University, Shenzhen, China

**Keywords:** Inflammation, Activated microglia, Ischemic stroke, Integrin αvβ3, TNF-α

## Abstract

Inflammation plays a pivotal role in ischemic stroke, when activated microglia release excessive pro-inflammatory mediators. The inhibition of integrin αvβ3 improves outcomes in rat focal cerebral ischemia models. However, the mechanisms by which microglia are neuroprotective remain unclear. This study evaluated whether post-ischemic treatment with another integrin αvβ3 inhibitor, the cyclic arginine-glycine-aspartic acid (RGD) peptide-cGRGDdvc (LXW7), alleviates cerebral ischemic injury. The anti-inflammatory effect of LXW7 in activated microglia within rat focal cerebral ischemia models was examined. A total of 108 Sprague-Dawley rats (250–280 g) were subjected to middle cerebral artery occlusion (MCAO). After 2 h, the rats were given an intravenous injection of LXW7 (100 μg/kg) or phosphate-buffered saline (PBS). Neurological scores, infarct volumes, brain water content (BWC) and histology alterations were determined. The expressions of pro-inflammatory cytokines [tumor necrosis factor-alpha (TNF-α) and interleukin-1 beta (IL-1β)], and Iba1-positive activated microglia, within peri-ischemic brain tissue, were assessed with ELISA, western blot and immunofluorescence staining. Infarct volumes and BWC were significantly lower in LXW7-treated rats compared to those in the MCAO + PBS (control) group. The LXW7 treatment lowered the expression of pro-inflammatory cytokines. There was a reduction of Iba1-positive activated microglia, and the TNF-α and IL-1β expressions were attenuated. However, there was no difference in the Zea Longa scores between the ischemia and LXW7 groups. The results suggest that LXW7 protected against focal cerebral ischemia and attenuated inflammation in activated microglia. LXW7 may be neuroprotective during acute MCAO-induced brain damage and microglia-related neurodegenerative diseases.

## Introduction

Ischemic stroke is the leading cause of mortality worldwide, with a high incidence of permanent disability in survivors (1). Ischemic stroke is caused by the interruption of blood flow to the brain, owing to an embolic or thrombotic occlusion. This initiates complex and extensive metabolic changes, including the release of reactive oxygen species and cytokines, and the up-regulation of adhesion molecules on endothelial cells. The metabolic changes result in rapid neuroinflammation and ultimately cause cell death (2). Post-ischemic inflammation has been increasingly recognized as a pivotal contributor to the pathophysiology of ischemic stroke.

Recent evidence has shown that cells of the immune system are intimate participants in all stages of the ischemic stroke cascade. Microglia constitute the innate immune cells of the central nervous system, and are rapidly activated after ischemic stroke, by changing shape and phenotype (3). Activated microglia have the potential to mediate beneficial activities, such as phagocytizing debris, but in the acute stages of ischemic stroke, they are thought to be a potential contributor to "secondary injury". This occurs via the production of excessive pro-inflammatory cytokines, such as tumor necrosis factor-alpha (TNF-α), interleukin-1 beta (IL-1β), and other pro-inflammatory mediators (4,5). Thus, the suppression of microglia-mediated excessive inflammatory responses during the acute stages of brain injury may prove to be a valid therapeutic strategy for ischemic stroke.

The inhibition of intercellular adhesion molecules, such as integrin αvβ3, for the treatment of ischemic stroke has drawn attention recently. Research has shown that integrin αvβ3 is up-regulated in ischemic brain lesions and mediates the activation of vascular endothelial growth factor (VEGF) receptors (6,7). The integrin αvβ3 receptor domain contains the peptide sequence arginine-glycine-aspartic acid (RGD), which can be inhibited by cyclo-RGDfV (8). Pre- and post-ischemic treatment with an inhibitor of integrin αvβ3 improves the outcomes of ischemic stroke in rats, via inhibition of the VEGF-mediated vascular permeability, and by reducing the infiltration of inflammation materials into ischemic lesions (9). More specifically, research has shown that αv integrins are important mediators of microglial activation (10).

The cyclic RGD peptide cGRGDdvc (LXW7) is another novel, low molecular weight peptide ligand that shows high targeting efficacy and specificity for the αvβ3 integrin, both *in vitro* and *in vivo* (11). In this study, we tested whether post-stroke treatment with LXW7 alleviates cerebral ischemic injury and examined the anti-inflammatory effects of LXW7 in activated microglia, within rat transient focal cerebral ischemia.

## Material and Methods

### Animals

This research project was carried out within an appropriate ethical framework. The handling and use of rats was approved by the Institutional Animal Care and Use Committee at Shenzhen PKU-HKUST Medical Center and procedures were conducted in compliance with the Health Guide for the Care and the Use of Laboratory Animals. All efforts were made to minimize the number of rats used and their suffering. Male Sprague-Dawley rats, weighing 250–280 g, were obtained from Guangdong Medical Laboratory Animal Center (Guangdong, China). The animals were randomly assigned to 3 treatment groups (n=36 per group): 1) MCAO with LXW7 treatment (100 μg/kg; LXW7 group); 2) sham operation (sham-operated group), and 3) MCAO with phosphate-buffered saline (PBS; Sangon Biotech Co., Ltd., China) treatment (ischemia group).

Stroke surgery was performed via transient middle cerebral artery occlusion (MCAO) using the intraluminal suture method, as previously described (12). Briefly, rats were anesthetized with an intraperitoneal injection of 50 mg/kg pentobarbital sodium. A silicon-coated 4–0 monofilament nylon suture was introduced into the right internal carotid artery, through the common carotid artery, to occlude the origin of the middle cerebral artery. After 2 h of occlusion, the suture was withdrawn to allow reperfusion for 24 h. Rectal temperature was maintained at 37±0.5°C with a heating pad throughout the surgical procedures. The rats for the sham operation were subjected to the surgical procedures without the suture for intraluminal occlusion.

### Drug administration

LXW7 was designed by the Department of Biochemistry and Molecular Medicine, University of California Davis, Sacramento, CA, USA. The dose used for the intravenous injection of LXW7 was derived from previous studies (9,13). Rats in the LXW7 group received an intravenous injection of LXW7 (100 μg/kg body weight) 2 h after MCAO was performed. The ischemia group received an intravenous injection of 0.5 mL PBS.

### Neurological evaluation and infarct assessment

Eighteen rats were used to assess neurological deficit before cerebral ischemia and 24 h after MCAO (n=6), by an investigator who was blind to the experimental conditions of the animals. The neurological evaluations were scored on a five-point scale, based on the Zea Longa scale (12): 0, no observable neurologic deficit; 1, failing to extend left forepaw fully; 2, circling to the left; 3, falling to the left; 4, no movement or spontaneous walking and a depressed level of consciousness. Brains were then harvested to assess the infarct volume, as described previously (14). Briefly, brains were sectioned at 2-mm intervals from the frontal pole using a rat brain matrix. Slices were incubated with 2% 2,3,5-triphenyltetrazolium chloride (TTC) for 15 min at 37°C. Images of the slices were digitized and the infarct areas were measured using Image J software (National Institutes of Health, NIH, USA). A correction for brain edema was made according to standard methods (contralateral hemisphere volume − volume of non-ischemic ipsilateral hemisphere) (15). The infarct volume is reported as a percentage of the contralateral hemisphere volume.

### Measurement of brain water content

Brains were removed rapidly and were dissected into hemispheres through the interhemispheric fissure. Brain tissues were weighed immediately (wet weight) and then dried at 110°C for 24 h. The water content (%) was calculated as: [(wet weight - dry weight)/wet weight] × 100.

### Western blot analysis

The peri-infarct brain tissues from each hemisphere were used for western blotting. Tissues were homogenized with protein extraction reagent (Pierce, USA) containing protease inhibitors and then centrifuged at 12,000 *g* for 10 min at 4°C. The protein concentration of the supernatant was measured (Bio-Rad, USA), and equivalent amounts of protein were loaded for SDS-PAGE. Protein bands were transferred to polyvinylidenedifluoride membranes and blocked with 5% non-fat milk for 1 h. The membranes were incubated overnight at 4°C with anti-VEGF (1:1000; Abcam, UK), anti-phosphorylated VEGF Receptor 2 (1:1500; Abcam), and anti-VEGF receptor 2 (1:1000; Abcam), rabbit TNF-α (1:1000, Proteintech Inc., China), IL-1β (1:500, R&D Systems Inc., USA), Iba1 (1:1000, Wako Pure Chemical Industries, Japan), and β-actin (1:3000, Bioworld Technology Inc., USA). Then, they were incubated with horseradish peroxidase conjugated second antibodies. The protein was detected with a chemiluminescence kit (ECL plus; Amersham Bioscience, USA). The densities of the bands were quantified in Image J software (National Institutes of Health). All data were corrected and normalized to β-actin.

### Enzyme-linked immunosorbent assay (ELISA) analysis

Protein levels of TNF-α and IL-1β in the peri-infarct brain tissues were also measured using ELISA kits (R&D Systems Inc.), according to the manufacturer's protocol. The intensity of the enzyme reaction was read within 30 min using an enzyme micro-plate at 450 nm. The mean value from two wells per rat was calculated.

### Histological analysis

Rats were perfused transcardially with saline followed by cold 4% paraformaldehyde in 0.1 M PBS. Brains were removed and fixed in 4% paraformaldehyde overnight at 4°C and then paraffin embedded. Serial coronal sections (5-µm thick) were cut on a microtome. Brain sections were treated with 3% H_2_O_2_ for 10 min and then blocked with 10% normal goat serum for 1 h at room temperature. Then, the sections were reacted with primary antibodies, including anti-VEGF (1:1000; Abcam), anti-phosphorylated VEGF Receptor 2 (1:400; Abcam), overnight at 4°C. Subsequently, the sections were incubated with anti-rabbit secondary antibody for 50 min at room temperature. The primary antibodies were replaced with PBS in the negative control analyses. The sections were exposed to 3,3′-diaminobenzidine tetrahydrochloride (DAB) reagent and counter-stained with hematoxylin.

Double immunofluorescence staining was performed to detect the expression of pro-inflammatory cytokines (TNF-α and IL-1β) in the microglia. Brain sections were incubated with 10% normal goat serum for 1 h at room temperature and then incubated in a humidified chamber with the primary antibodies Iba1 (1:500, Wako Pure Chemical Industries), IL-1β (1:100, R&D Systems Inc.), and TNF-α (1:50, Proteintech Inc.) overnight at 4°C. The sections were rinsed with cold PBS and incubated with the fluorescent secondary antibodies Alexa Fluor¯ 488 goat anti-mouse IgG (H+L) (1:1000; Cell Signaling Technology, USA) and Alexa Fluor¯ 555 goat anti-rabbit IgG (1:1000; Cell Signaling Technology) for 1 h at room temperature. Following rinsing in PBS, fluorescent mounting medium was applied to the sections. Co-localization was observed by confocal microscopy (LSM5 PASCAL; Carl Zeiss, Germany).

### Statistical analysis

Data are reported as means±SD. Statistical significance was analyzed using one-way analysis of variance (ANOVA), followed by the Fisher's *post hoc* test. P<0.05 was considered to be statistically significant. SPSS 18.0 statistical software (USA) was used to analyze the data.

## Results

### LXW7 treatment decreased p-Flk-1 and VEGF expression

**Figure 1:**
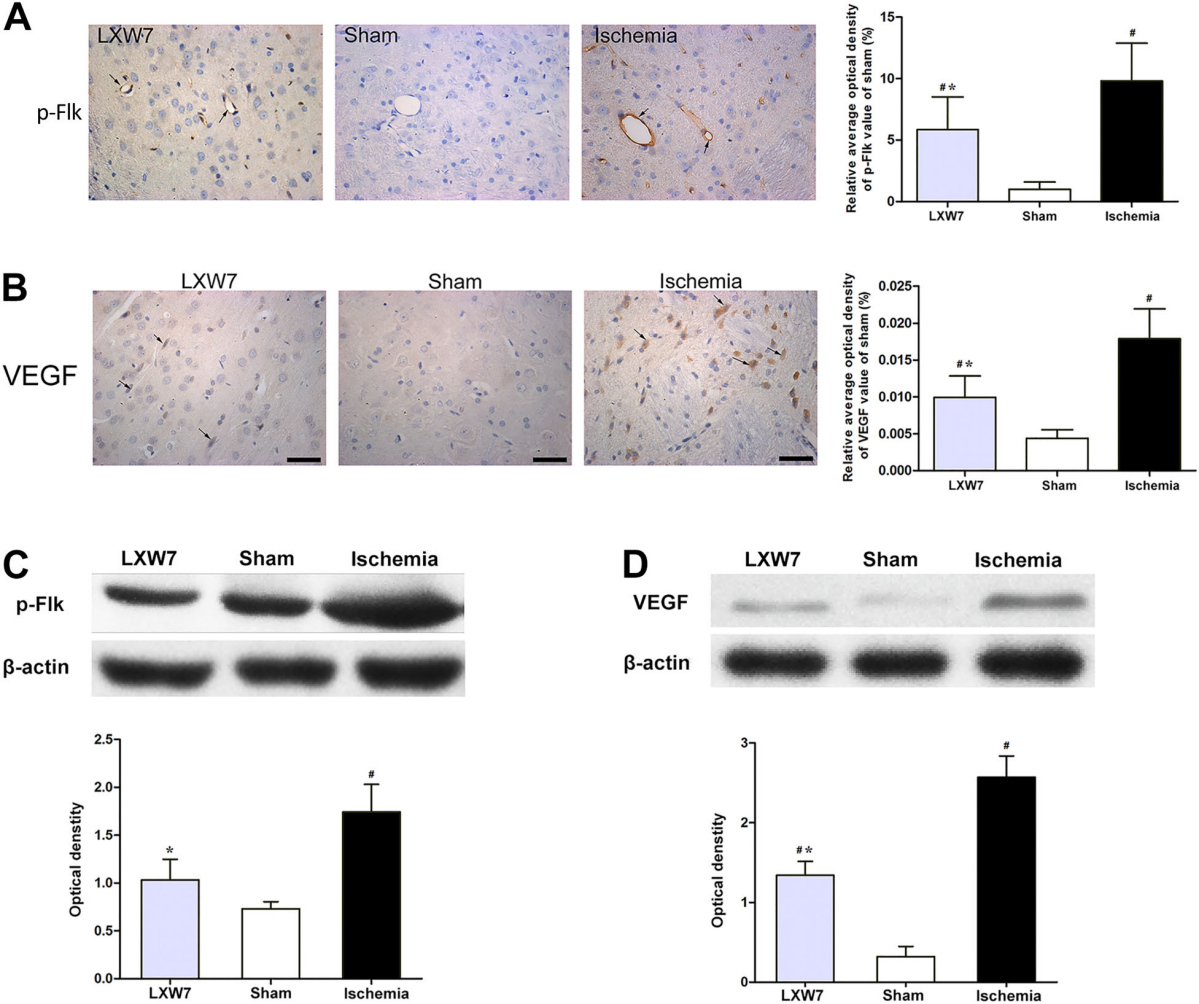
Effect of LXW7 on P-Flk (*A*, *C*) and vascular endothelial growth factor (VEGF) expression (*B*, *D*) in rats with middle cerebral artery occlusion (MCAO) treated with LXW7 (LXW7 group), PBS (ischemia group) and sham-operated rats (sham group) at 24 h. Arrowheads indicate positive cells in the brain tissue. Scale bar: 50 μm. Data are reported as means±SD (n=6 for each group). ^#^P<0.05 compared to sham group, *P<0.05 compared to ischemia group (ANOVA, followed by Fisher's *post hoc* test).

Western blot analysis and immunohistochemical staining revealed that 24 h after the ischemic injury the expression of p-Flk-1 was significantly higher in the ischemia group compared to the sham-operated group. LXW7 treatment markedly reduced the ischemia-induced elevation of p-Flk-1 (P<0.05; Figure 1A and C), one of the receptors of the VEGF.

The expression of VEGF was also significantly elevated in the ischemia group, compared to the sham-operated group, according to western blot analysis (P<0.05). The LXW7 group had lower VEGF expression, compared to the ischemia group (Figure 1D). Moreover, immunostaining of the VEGF was also lower in the LXW7 group, when compared to the ischemia group (P<0.05: Figure 1B).

### LXW7 treatment did not affect the neurological deficit score, but did ameliorate brain edema, and decreased infarct size

**Figure 2:**
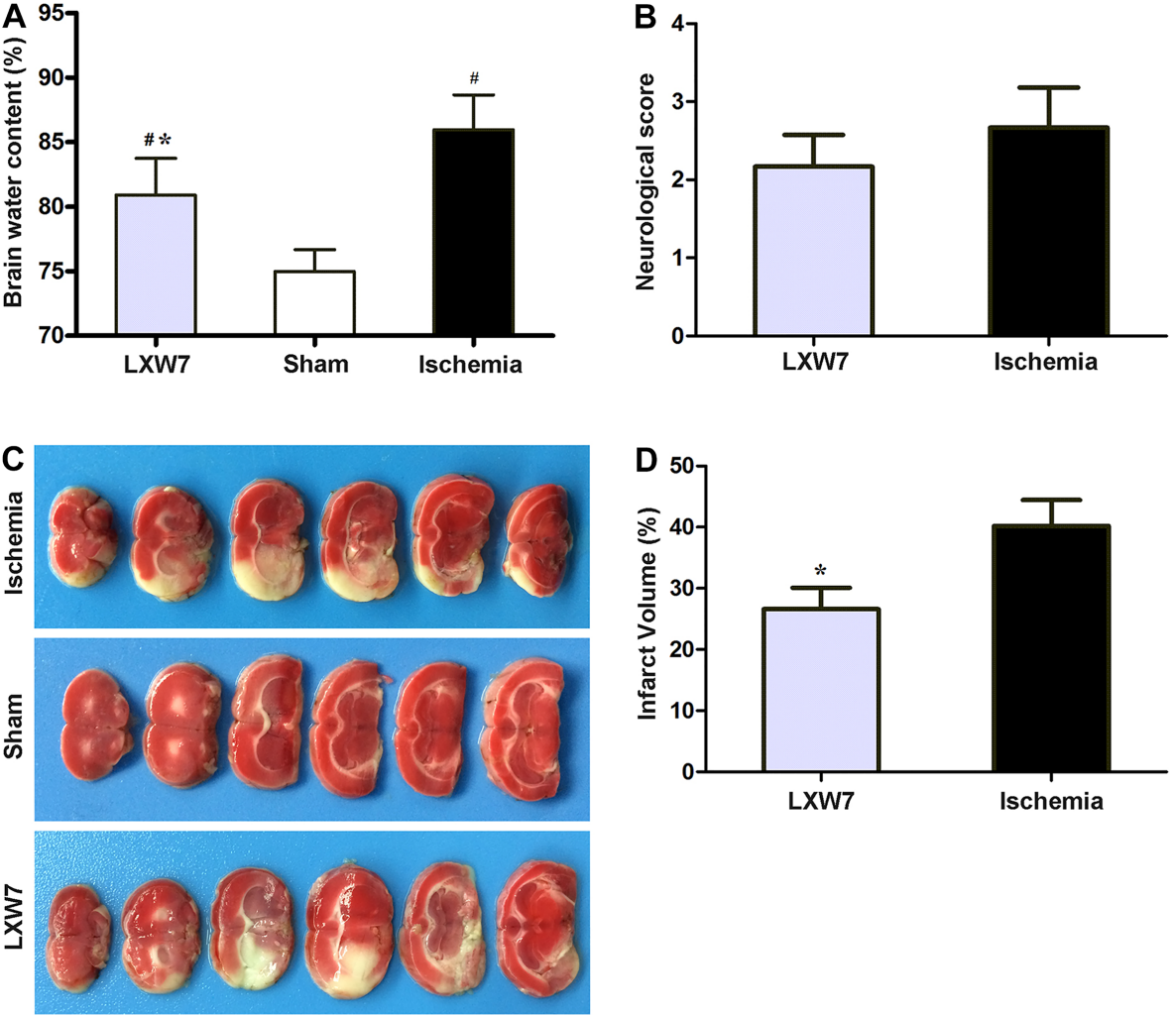
Effect of LXW7 on brain water content (*A*), neurological deficits (*B*), and brain infarct volume (*C*, *D*) in rats with middle cerebral artery occlusion (MCAO) treated with LXW7 (LXW7 group), PBS (ischemia group) and sham-operated rats (sham group) at 24 h. Data reported as means±SD (n=6 for each group). ^#^P<0.05 compared to sham group, *P<0.05 compared to ischemia group (ANOVA, followed by Fisher's *post hoc* test).

There was no difference in the Zea Longa scores between the ischemia group and the LXW7 group 24 h after ischemic injury (P<0.05; Figure 2B).

The water content of the ipsilateral cerebral hemisphere was significantly higher 24 h after ischemia in the ischemia group, compared to the sham-operated group (P<0.05) (Figure 2A). In comparison with the ischemia group, significantly lower water contents were observed in the LXW7 group (P<0.05; Figure 2A). The TTC staining images showed that the ischemia group had obviously higher infarct volumes compared to the sham-operated group (Figure 2C) and the LXW7 group had significantly lower infarct volumes compared to the ischemia group (P<0.05; Figure 2C and D).

### LXW7 treatment suppressed the expression of inflammatory cytokines

**Figure 3:**
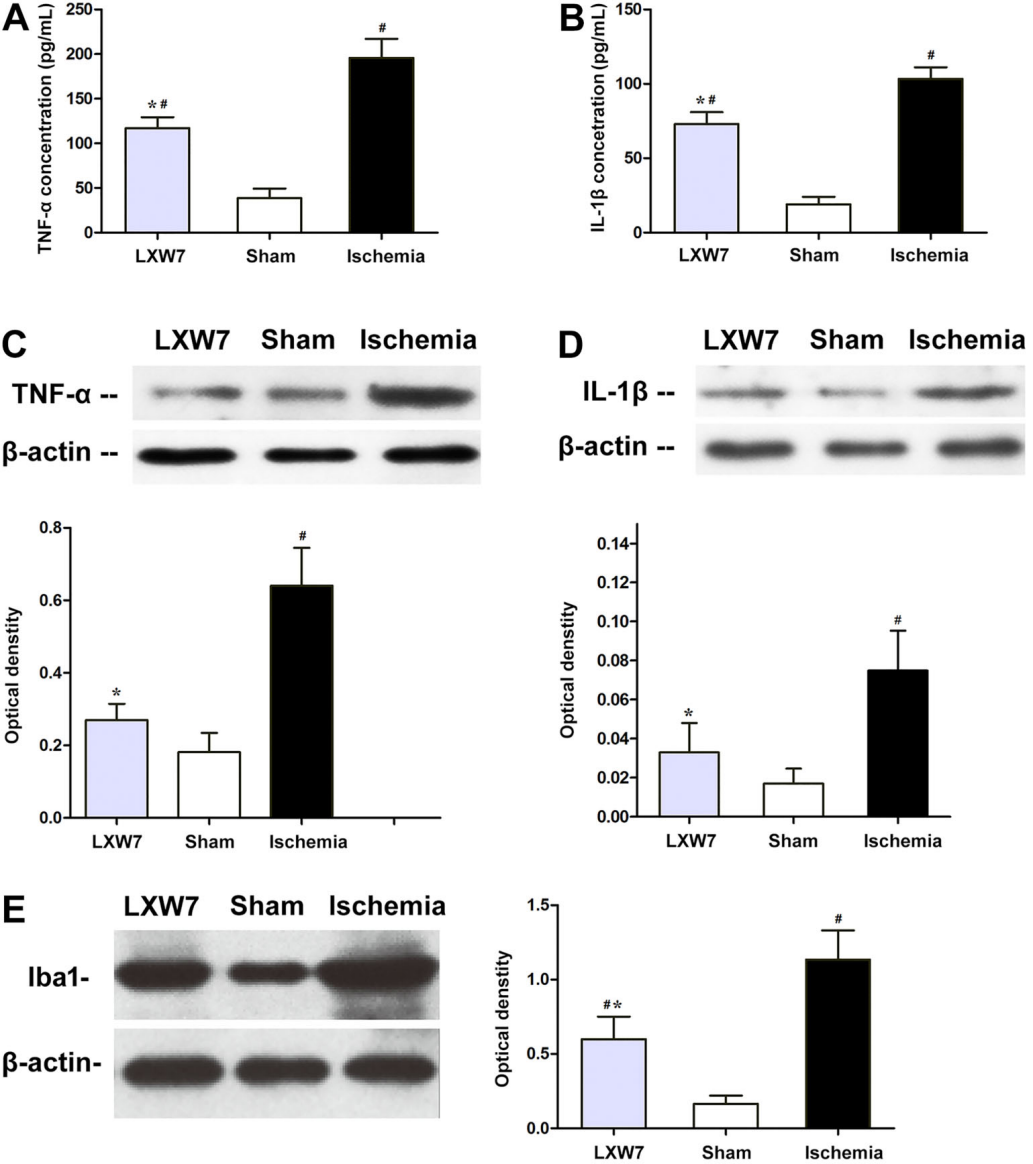
Protein levels for proinflammatory cytokines TNF-α, IL-1β, and marker of microglia Iba1 in the peri-ischemic brain tissue 24 h after middle cerebral artery occlusion in rats treated with LXW7 (LXW7 group), PBS (ischemia group) and sham-operated rats (sham group). Expression of TNF-α is shown in *panel A*, and IL-1β in *panel B,* by ELISA analysis. Representative western blots are shown in *panels C*, *D*, and *E.* Data are reported as means±SD (n=6 for each group) and corrected by β-actin. ^#^P<0.05 compared to sham group, *P<0.05 compared to ischemia group (ANOVA, followed by Fisher's *post hoc* test).

Excessive production of pro-inflammatory cytokines contributes to ischemic brain damage. The expressions of the TNF-α and IL-1β proteins were significantly higher in the ischemia group, compared to the sham-operated group (P<0.05; Figure 3C-D). The LXW7 group exhibited lower expressions of TNF-α and IL-1β, compared to the ischemia group (P<0.05; Figure 3C-D).

**Figure 4 f04:**
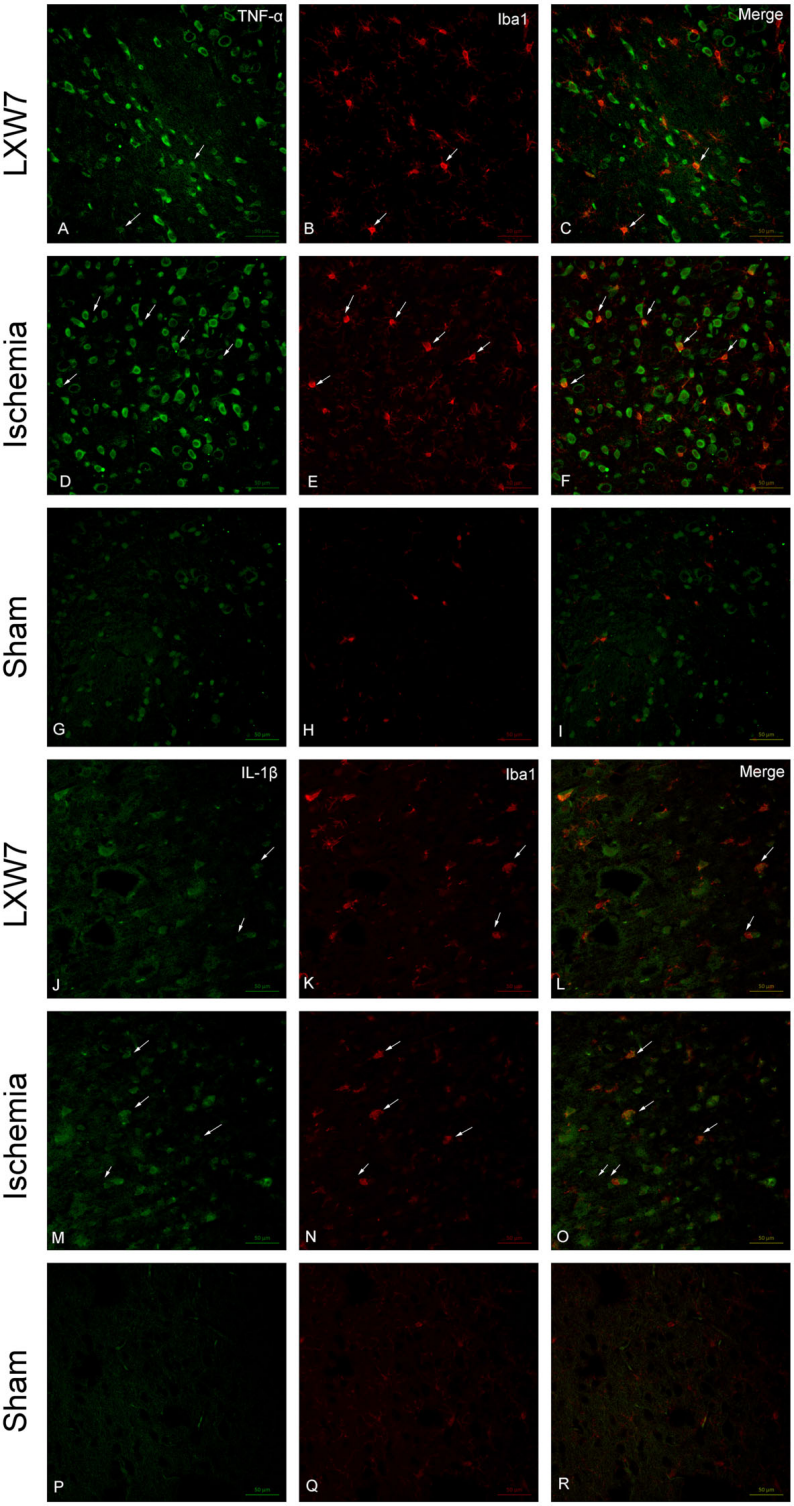
Treatment of middle cerebral artery occlusion rats with LXW7 (LXW7 group) resulted in the reduction of TNF-α, and IL-1β expression in activated microglia. Confocal images showing the expression of TNF-α (*A*, *D*, *G,* green), IL-1β (*J*, *M*, *P*, green) in microglia (*B, E*, *H*, *K*, *N*, *Q*, red), in peri-ischemic brain tissue 24 h after MCAO in rats (*D*-*F*, M-O) and following treatment with LXW7 (*A*-*C*, *J*-*L*) (n=6 for each group). Expression of TNF-α and IL-1β in microglia cells (arrows) is markedly enhanced following MCAO, but a noticeable reduction in TNF-α and IL-1β expression can be observed in the activated microglia in the LXW7-treated rat brain. Activated microglia were reduced with LXW7 treatment (*B*, *K*). Scale bars: 50 µm.

The expression levels of TNF-α and IL-1β in the peri-ischemic brain tissue were also measured with ELISA. Compared to the sham-operated group, both TNF-α and IL-1β were markedly higher after ischemic injury in the ischemia group (P<0.05; Figure 3A and B); however, treatment with LXW7 suppressed the expression of TNF-α and IL-1β, by 29 and 39%, respectively, compared to the ischemia group (P<0.05; Figure 3A and B).

### Cellular localization of TNF-α and IL-1β in activated microglia in peri-ischemic brain tissue

Iba1 antibody is a specific microglial marker and is up-regulated during the activation of microglia. Western blot showed that the microglia marker Iba1 concentrations were significantly higher in the peri-infarct cortical tissues from the ischemia group, compared to the sham-operated group. Compared to the ischemia group, there were markedly lower Iba1 concentrations in the LXW7 group. Activated microglia are defined as the cells that have undergone morphological change into their activated ameboid phenotype. To study the anti-inflammatory effects of LXW7 in the activated microglia, we examined the expression of the pro-inflammatory cytokines, including TNF-α and IL-1β in the activated microglia within peri-ischemic brain tissue, using double immunofluorescence staining. The confocal images showed that the expression of TNF-α and IL-1β in the activated microglia was significantly higher 24 h after the ischemic injury, compared to the sham-operated group, but it was markedly lower after treatment with LXW7 (compared to the ischemia group). Furthermore, immunofluorescence staining also showed a reduction of activated microglia in the LXW7 group.

## Discussion

Endothelial cells express several integrin heterodimers, including αvβ3, α5β1 and αvβ5. Among these, integrin αvβ3 is crucial for mediating the activation of VEGF receptors, and the anti-integrin αvβ3 antibody inhibits phosphorylation of the VEGF receptors (16). Flk-1 is one of the VEGF receptors, a main mediator of the angiogenic and permeability-enhancing effects of VEGF, and is activated in a phosphorylated form (17). VEGF expression is up-regulated after the onset of ischemia; over-expression of VEGF has a deleterious effect during the initial period of brain ischemia, causing blood-brain barrier disruption, brain edema, and hemorrhagic transformation, which often comprise the fatal complications of strokes (18). Prior research has shown that the inhibition of integrin αvβ3 improves the outcomes of ischemic stroke in rats, through the inhibition of VEGF-mediated vascular permeability (9). In the present study, we examined the post-ischemic effect of the cyclic RGD peptide, GRGDdvc (LXW7; another novel inhibitor of integrin αvβ3), on focal cerebral ischemia in rats. Post-ischemic treatment with LXW7 decreased the infarction volume, and ameliorated brain edema. In addition, western blot analysis and immunohistochemical staining revealed that the expressions of P-Flk-1 and VEGF in the LXW7 treatment group were lower, which is consistent with previous reports (9) and suggests a protective effect of LXW7 on ischemic brain injury.

Inflammation is a crucial contributor in the ischemic cascade after cerebral ischemia that results in harmfully augmenting "secondary injury". Microglia are activated within minutes of the onset of focal cerebral ischemia and trigger an innate immune response, undergoing phenotypic transformation to become "ameboid". Activated microglia can damage brain neurons through the excessive release of pro-inflammatory mediators, including reactive oxygen species and inflammatory cytokines, which damage endothelial cells and result in post-ischemic blood-brain barrier disruption (19,20). A direct effect on inflammation is supported by research showing that post-ischemic treatment with microglial inhibitors (21,22) suppressed microglial activation and the expression of pro-inflammatory mediators, and attenuated ischemic brain injury. In addition, antibodies against TNF-α and IL-1β treatment have been shown to reduce ischemia brain damage (23,24). Previous studies have shown that the inhibitor of integrin αvβ3 reduced the transmigration of monocytes or macrophages through vessel walls and decreased the infiltration of inflammatory cells into cerebral ischemia lesions (9,25). Milner et al., using flow cytometry, revealed that microglia expressed the αvβ3 integrin, in addition to αvβ5, and they are regulated by cytokines. Fibronectin and vitronectin induce microglial activation via the integrins α5β1 and αvβ5, respectively (26). Furthermore, Jin et al. found that RGD-containing icosamer OPN peptide (OPNpt20) confers anti-inflammatory effect in primary microglia cultures, which was obtained by αvβ3 integrin mediated suppression of iNOS induction, indicating the critical roles of αvβ3 integrin in mediating the physical and functional cell-cell or cell-matrix interactions (27).

We have extended the understanding from previous research by examining the anti-inflammatory effect of LXW7 in activated microglia within the cerebral ischemia model. Our results revealed that treatment with LXW7 inhibited the production of TNF-α and IL-1β in peri-ischemic brain tissue 24 h after ischemic injury. We also showed that early treatment with LXW7 markedly reduced TNF-α and IL-1β protein levels in the activated microglia. Furthermore, the microglia marker Iba1 was significantly higher in the peri-ischemic brain tissue after ischemic injury (according to the western blot analysis), suggesting that ischemia results in excessive microglial activation; but the level of Iba1 expression in the LXW7-treated group was lower. In addition, immunofluorescence staining of the peri-ischemic brain tissue demonstrated that LXW7 treatment decreased the activation of microglia.

These data indicate that the neuroprotective effects of LXW7 involve an inhibition of VEGF-mediated vascular permeability. It may also be associated with the attenuation of inflammatory responses via decreasing microglia activation and reducing the release of pro-inflammatory cytokines in ischemic brain tissue. This anti-inflammatory effect may have several mechanisms: i) the correlation with the regulatory effects of αvβ3 integrin on activated microglia, and ii) the association with lower expression of VEGF. VEGF is known to have pro-inflammatory effects and enhance macrophage activity (28). Brain ischemia leads to the activation of microglia by increasing the levels of cytokines, glutamate and heat shock protein 70 (HSP70) (29); lower expression levels of VEGF may prevent the inflammatory response in cerebral ischemia lesions and decrease microglial activation. Moreover, previous studies showed that inhibitors of integrin αvβ3 therapy were associated with decreased fibrinogen/fibrin deposition (30), and fibrin deposition mediates neuro-inflammatory effects, as an activator of microglia during stroke. However, we did not study this interaction here. In this study, there was no difference in the Zea Longa scores between the ischemia group and the LXW7 group 24 h after ischemic injury. There are several possible explanations for this absence of neurological deficit reduction: i) the small sample in our experiment for neurological deficit evaluation may have contributed to inaccurate verification; ii) the limitation of using the Zea Longa scale, the staple assessment for motor disturbances. More rigorous tests should be performed in further studies. In addition, a delayed improvement in neurological outcome, or possible inaccuracies made by the investigator should be taken into consideration. Various cyclic peptides have shown a high binding affinity to integrin αvβ3, and are currently being tested in clinical trials for the treatment of different cancers (31), but have rarely been considered in cerebrovascular disease research. In the present study, LXW7, a low molecular weight peptide ligand, showed high targeting efficacy and specificity against the αvβ3 integrin, and high tumor uptake, but low liver uptake, both *in vivo* and *ex vivo*; it exerted a neuroprotective effect in the MCAO model. However, it should be noted that this study examined only the role of microglia activation in ischemic brain injury. Previous studies have shown that astrocytes are also activated in cerebral ischemia and they release several kinds of pro-inflammatory cytokines (32,33). Further studies should be performed to evaluate the relationship between activated microglia and astrocytes during LXW7 treatment after ischemic stroke. Besides this, the exact mechanisms by which LXW7 regulates the activation of microglia need to be defined.

In conclusion, this study demonstrated that the administration of LXW7 decreased infarct volume and ameliorated brain edema. In addition, the neuroprotective effects of LXW7 may be associated with the attenuation of microglia activation and reduction in the release of pro-inflammatory cytokines in ischemic brain tissue. LXW7 may prove to be a valid potential therapy for acute cerebral ischemic injury and microglia-related diseases.
